# The c-Rel transcription factor limits early interferon and neuroinflammatory responses to prevent herpes simplex encephalitis onset in mice

**DOI:** 10.1038/s41598-021-00391-7

**Published:** 2021-10-27

**Authors:** Mathieu Mancini, Benoît Charbonneau, David Langlais, Silvia M. Vidal

**Affiliations:** 1 Department of Human Genetics, McGill University, Montreal, QC Canada; 2 McGill University Research Centre on Complex Traits, McGill University, Montreal, QC Canada; 3 McGill University Genome Centre, Montreal, QC Canada; 4 Department of Medicine, McGill University, Montreal, QC Canada

**Keywords:** Computational biology and bioinformatics, Genetics, Immunology

## Abstract

Herpes simplex virus type 1 (HSV-1) is the predominant cause of herpes simplex encephalitis (HSE), a condition characterized by acute inflammation and viral replication in the brain. Host genetics contribute to HSE onset, including monogenic defects in type I interferon signaling in cases of childhood HSE. Mouse models suggest a further contribution of immune cell-mediated inflammation to HSE pathogenesis. We have previously described a truncating mutation in the c-Rel transcription factor (*Rel*^*C307X*^) that drives lethal HSE in 60% of HSV-1-infected *Rel*^*C307X*^ mice. In this study, we combined dual host-virus RNA sequencing with flow cytometry to explore cell populations and mechanisms involved in *Rel*^*C307X*^-driven HSE. At day 5 postinfection, prior to HSE clinical symptom onset, elevated HSV-1 transcription was detected together with augmented host interferon-stimulated and inflammatory gene expression in the brainstems of high-responding *Rel*^*C307X*^ mice, predictive of HSE development. This early induction of host gene expression preceded pathological infiltration of myeloid and T cells in *Rel*^*C307X*^ mice at HSE onset by day 7. Thus, we establish c-Rel as an early regulator of viral and host responses during mouse HSE. These data further highlight the importance of achieving a balanced immune response and avoiding excess interferon-driven inflammation to promote HSE resistance.

## Introduction

Herpes simplex encephalitis (HSE) is primarily caused by infection of the central nervous system (CNS) with herpes simplex virus 1 (HSV-1). With an estimated incidence of 2–4 per 100,000 individuals per year, HSE is the most common form of sporadic viral encephalitis^1^. Approximately one third of HSE cases occur in children, while the average age of onset for adult HSE is 60 years of age^2^. HSE typically involves focal inflammatory lesions in the frontal or temporal lobes, or rarely, inflammation of the brainstem^3,4^. Genetic factors are known to contribute to HSE onset and severity in children, namely, single gene defects in the Toll-like receptor 3 (TLR3) cascade including *TLR3*, *UNC93B1*, *TRIF*, *TRAF3*, *TBK1*, *IRF3* and *IFNAR1*^5–11^. These mutations result in defective type I interferon (IFN) production, where for example *TLR3* deficient patient-derived neurons and oligodendrocytes exhibit compromised cell-intrinsic control of HSV-1 infection^12^. However, these TLR3/type I IFN-related variants only explain a minority of childhood HSE cases. To date, other genetic etiologies unrelated to type I IFN have also been identified, including the RNA lariat debranching enzyme *DBR1* gene in childhood cases, the *MASP2* gene reported in adult HSE patients and involved in the innate immune complement system^13,14^, and the small nucleolar RNA-encoding *SNORA31* gene identified in five HSE patients of various ages and involved in the neuronal antiviral response^15^.

Single gene knockout studies in mouse models of HSV-1 infection have confirmed that type I IFN and IFN-stimulated gene (ISG) expression downstream of pathogen sensing is generally protective in the brain^16^. However, excessive IFN-mediated responses can also be detrimental in the brain and underlie various interferonopathies in mice and humans^17,18^. This tightly regulated balance between beneficial and damaging host responses has been further explored in the context of the immune cell-mediated response to mouse HSE. Resident CX3CR1^+^ microglia, involved in tissue maintenance, proinflammatory cytokine secretion, and expression of chemokines, are protective during mouse HSE^19–21^. The cytotoxic and IFN-γ secreting functions of invading NK cells, CD4^+^, and CD8^+^ T cells are also essential to controlling HSV-1 viral replication in the CNS^22,23^. However, chemokine receptor deficient *Cxcr3*^*−/−*^ and *Ccr5*^*−/−*^ mice are resistant to HSE, suggesting that lymphocyte and myeloid infiltration may contribute to pathological neuroinflammation^24–26^. Recently, invading Ly6C^+^ monocytes and Ly6G^+^ neutrophils have also been shown to exacerbate lethal HSE in mice^27,28^. Thus, the genetic regulation of cell-mediated inflammation, like type I IFN, is an important determinant of HSE in mice.

In a previous study, we identified a novel mutation in the reticuloendotheliosis oncogene *Rel*, encoding the NF-κB family transcription factor subunit c-Rel, that caused HSE susceptibility^29^. Homozygous *Rel*^*C307X*^ mice express a truncated c-Rel product deprived of its transactivation domains, and upon intranasal (i.n.) HSV-1 infection experience a 60% decrease in survival between days 6 and 9 postinfection (p.i.) compared to resistant wild-type *Rel*^+*/*+^ mice. Interestingly, *Rel*^*C307X*^ mice are not deficient in their cell-intrinsic type I IFN response to infection. Rather, HSE susceptibility in mutant animals is cell-mediated, and requires the expression of the *Rel*^*C307X*^ variant in both hematopoietic and non-hematopoietic cells. Thus, defects in c-Rel-dependent regulation of infiltrating immune cells and of brain-resident cells, together, lead to increased viral load, neuroinflammation, and caspase-3-dependent cell death in moribund *Rel*^*C307X*^ mice.

In the present study, to determine which c-Rel-dependent mechanisms and cell types are involved in HSE development in the *Rel*^*C307X*^ CNS, we performed dual virus and host gene expression profiling in the brainstem of HSV-1 infected *Rel*^*C307X*^ and *Rel*^+*/*+^ mice prior to disease onset. Augmented viral gene transcription was detected as early as day 5 p.i. in 3/6 *Rel*^*C307X*^ brainstems, together with elevated interferon-related inflammatory gene expression that contributed to pathological T cell and myeloid recruitment by day 7. Furthermore, the early induction of viral and host gene expression in high-responding *Rel*^*C307X*^ mice at day 5 p.i. was detected at least one full day before the expected onset of HSE symptoms in susceptible mice, and may be predictive of later HSE severity and outcome. Thus, c-Rel is protective during HSV-1 infection of the brainstem.

## Results

### ***Rel***^***C307X***^ mice exhibit differential viral and host responses as early as day 5 in HSV-1-infected brainstems

We have previously demonstrated that *Rel*^*C307X*^ mutant mice are susceptible to HSE, where over half of *Rel*^*C307X*^ mice exhibit high viral titers and reach clinical endpoint between days 6 to 9 post-HSV-1 infection, compared to resistant *Rel*^+*/*+^ littermates^29^. Here, to determine the role of the *Rel*^*C307X*^ variant in the development of HSE, we performed dual RNA-sequencing (RNA-seq) in HSV-1-infected *Rel*^+*/*+^ and *Rel*^*C307X*^ littermate mice. We focused on the brainstem, which during experimental mouse HSE is a well-characterized site of viral replication and contains varied resident and infiltrating hematopoietic cell types at the height of the disease^29,30^; in our model, wild-type c-Rel protein expression was detected in resident microglia and neurons as well as infiltrating lymphocytes and myeloid cells, both at steady-state and at day 5 post-HSV-1 infection (Supplementary Fig. 1). In this study, we also sought to detect early *Rel*^*C307X*^-dependent effects on host gene expression before the onset of fulminant HSE which had the potential to mask any causal effects of the mutation. Therefore, day 5 p.i. was selected as an optimal timepoint prior to the rapid onset of HSE clinical symptom and of pathological cell death in susceptible *Rel*^*C307X*^ mice.

**Figure 1:**
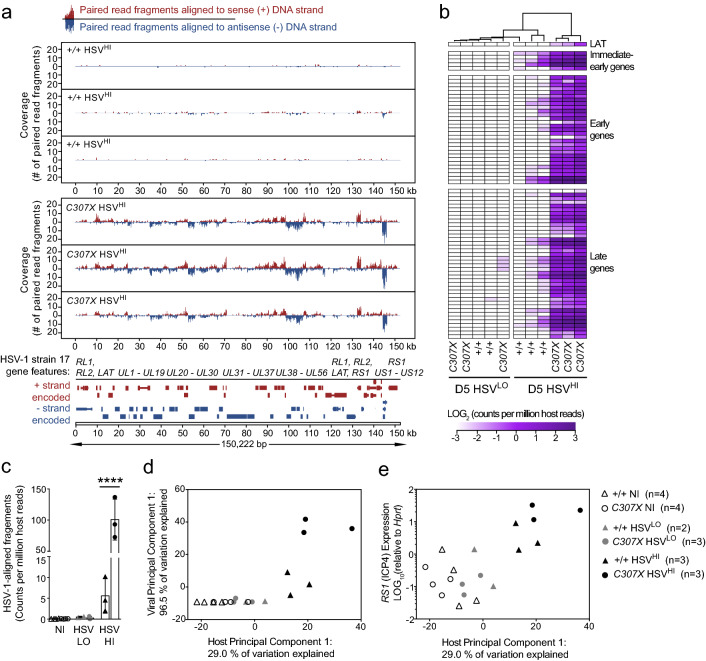
Global transcriptional changes in HSV-1-infected brainstems define high and low response groups among *Rel*^+*/*+^ and *Rel*^*C307X*^ mice. Dual RNA-seq was performed on brainstems collected from HSV-1-infected *Rel*^+*/*+^ (*n* = 5) and *Rel*^*C307X*^ (*n* = 6) female mice at day 5 p.i., along with corresponding non-infected controls (NI; *n* = 4 per genotype). (**a**) Coverage of viral transcript reads mapping to the sense or anti-sense strands of the HSV-1 strain 17 genome. 3 of 5 *Rel*^+*/*+^ and 3 of 6 *Rel*^*C307X*^ HSV-1-infected samples that demonstrated the highest coverage are denoted +*/*+ HSV^HI^ and *C307X* HSV^HI^. (**b**) Hierarchical clustering of HSV-1-infected samples into high- and low-responding groups [HSV^HI^ or HSV^LO^] based on the detection of 75 viral open reading frames. These 75 viral transcripts are grouped by their known expression kinetics, namely the immediate-early, early, and late gene phases, and the latency-associated transcript (LAT). (**c**) Total paired sequence fragments aligning to the HSV-1 genome per group, expressed as counts normalized per million host reads. (**d**,**e**) Separate principal component analyses (PCA) for all 19 samples were performed across all 75 viral transcripts, and across 16,279 host genes expressed at > 3 CPM in at least 3 samples. In (**d**) viral PC1 is shown against host PC1, and in (**e**) *RS1 *(*ICP4*) relative expression is shown against host PC1. Data represent mean ± SD in (**c**). Statistical tests were performed using a two-way ANOVA with Tukey’s multiple correction test in (**c**), ****p < 0.0001.

At day 5 p.i., sequences derived from HSV-1-encoded viral mRNA were detected in 3 of 6 *Rel*^*C307X*^ brainstems and in 3 of 5 *Rel*^+*/*+^ brainstems. For both groups, these mice were denoted as high-responders to infection, with the notable observation that *Rel*^*C307X*^ mice harboured higher levels of HSV-1 transcription compared to *Rel*^+*/*+^ mice (Fig. 1a). In the remaining low-responding mice (3 of 6 *Rel*^*C307X*^ and 2 of 5 *Rel*^+*/*+^, 16 or fewer paired read fragments had aligned to the HSV-1 genome, and fewer than 3 reads in non-infected mice (Supplemental Data File 1). Viral gene expression, consistently across 75 major open reading frames and three phases of infection^31^, was also more elevated in high-responding *Rel*^*C307X*^ mice (Fig. 1b). Furthermore, all three high-responding *Rel*^*C307X*^ mice were clearly segregated together following a principal component analysis across expressed HSV-1 genes (Supplementary Fig. 2a,b). Overall, in high-responding groups at day 5 p.i., the total number of coding region-aligned viral reads was more elevated in *Rel*^*C307X*^ mice compared to *Rel*^+*/*+^ mice (Fig. 1c). Thus, *Rel*^*C307X*^-driven differences in HSV-1 viral RNA transcription were detected by day 5 p.i. in the brainstem, at least one day prior to expected HSE clinical symptom onset.

To better determine if early and augmented detection of HSV-1-derived sequences could predict disease outcome and help refine downstream analyses, the expression of host-derived genes was also subjected to a principal component analysis (Supplementary Fig. 2c,d). To best define clusters of similarly-responding mice, the first host principal component was plotted against the first viral principal component (Fig. 1d), or against the relative expression of the *RS1 (ICP4)* viral gene measured by RT-qPCR (Fig. 1e), which both distinguished high-responders from low-responders and from non-infected mice. Animals with the most replicating virus also demonstrated the most polarized host responses in the brainstem, suggesting that high-responders, and especially those carrying the *Rel*^*C307X*^ mutation, may go on to develop more severe HSE disease. Ultimately, these findings informed the separation of sample groups by their genotype and by their response level to infection for the following transcriptome analyses.

### Altered cell survival and homeostatic responses in non-infected *Rel*^*C307X*^ brainstems

**Figure 2:**
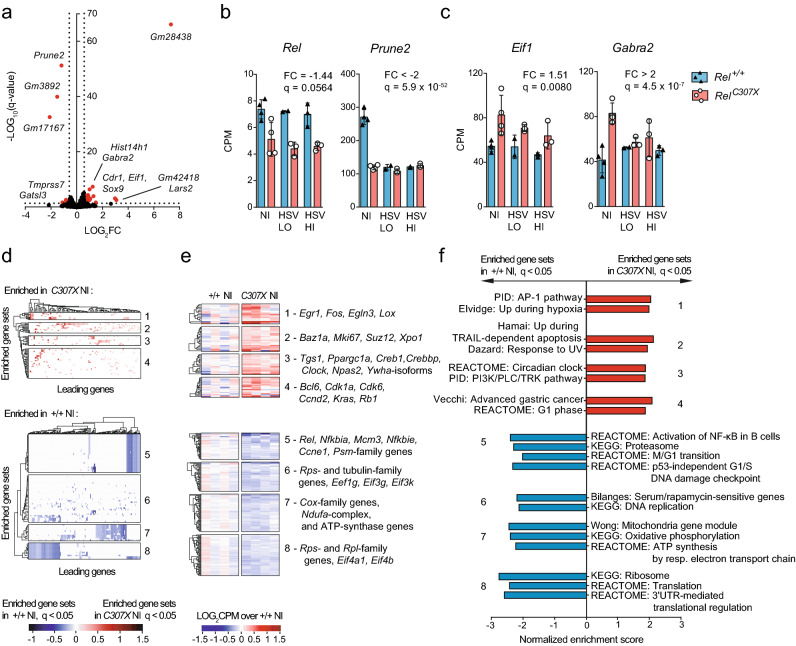
Differentially expressed gene networks in non-infected *Rel*^*C307X*^ brainstems. (**a**) Volcano plot of gene expression differences at steady-state in *C307X* NI compared directly to +/+ NI (*n* = 4 mice per group). Coloured points indicate differentially expressed genes (36 DEG upregulated, 9 DEG downregulated) with ≥ 1.5-fold change in expression and that met a threshold of *q* < 0.05 (BH-adjusted). (**b**,**c**) Expression of select *Rel*^*C307X*^-downregulated (**b**) and *Rel*^*C307X*^-upregulated (**c**) genes identified in (**a**) across all sample groups. (**d**) Hierarchical clustering of leading genes that drive the enrichment (*q* < 0.05) of curated gene sets in *C307X* NI mice (red, *N* = 66) and in +*/*+ NI mice (blue, *N* = 80) using GSEA. (**e**) Normalized expression of leading genes common to at least 10% of the enriched gene sets in each cluster defined in (**d**), with select genes highlighted for each cluster. (**f**) Representative enriched gene sets representative of *C307X* NI (red) and of +*/*+ NI (blue) enrichment clusters defined in (**d**). Data represent mean ± SD in (**b**,**c**). FC and *q* values (BH-adjusted) were assessed between NI groups using edgeR in (**b**,**c**). *q* values (BH-adjusted) and normalized enrichment scores were assessed using GSEA in (**d–f**).

To establish how the *Rel*^*C307X*^ mutation might disrupt gene expression in the brainstem at steady-state, non-infected *Rel*^+*/*+^ and *Rel*^*C307X*^ mice were directly compared to identify 45 differentially expressed genes (DEG) (Fig. 2a, Supplementary Fig. 3a). Of note, *Rel* is expressed in the brainstem, and is marginally downregulated in *Rel*^*C307X*^ mice at steady state (*q* = 0.0564, fold change = − 1.44), and significantly downregulated later during infection (*q* < 0.05, fold change < − 1.5), compared to wild-type mice (Fig. 2b). Other genes that were downregulated in *Rel*^*C307X*^ mice include *Prune2*, a tumour-suppressor gene with pro-apoptotic function^32^. On the other hand, *Gabra2*, involved in neural development and signaling^33^, and eukaryotic initiation factor *Eif1* were examples of upregulated genes in *Rel*^*C307X*^ brainstems (Fig. 2c). For a wider look at dysregulated pathways and functions, gene set enrichment analysis (GSEA) was performed across all brainstem-expressed genes, and enriched gene sets were grouped according to common leading-edge genes to better define overall up- or downregulated signatures in *Rel*^*C307X*^ mice (Fig. 2d–f).

First, 66 curated gene sets were enriched at a threshold of *q* < 0.05 in *Rel*^*C307X*^ mice (Fig. 2d–f, upper panels). In particular, those gene sets (Clusters 1 and 2) related to hypoxia, to the AP-1 transcription factor pathway, and to TRAIL- or UV-dependent apoptotic responses indicated that cell survival may be adversely impacted in *Rel*^*C307X*^ brainstems. Furthermore, other signatures related to cell growth and proliferation (Clusters 3 and 4) were over-represented in mutant samples, namely the phosphoinositide 3-kinase (PI3K) pathway, modulation of circadian rhythm (*Clock*, *Npas1*) and the G1 phase of cell division (*Rb1, Ccnd2, Cdk6, Bcl6*).

Second, among 80 enriched gene sets in *Rel*^+*/*+^ brainstems (Fig. 2d–f, lower panels), a distinct group of genes, including *Rel* and other NF-κB-family genes, underscored later stages of the cell cycle and the proteasome pathway (Clusters 5 and 6). Wild-type-enriched signatures also evoked mitochondrial function and cellular respiration, in addition to active transcription of ribosomal protein genes and heightened protein translation (Clusters 7 and 8). Altogether, these steady-state differences between *Rel*^+*/*+^ and *Rel*^*C307X*^ mice support a role for c-Rel in regulating cellular proliferation, homeostasis, and survival pathways in the brainstem.

Finally, we determined if any genes were up- or downregulated in *Rel*^*C307X*^ mice across all timepoints, independent of viral load or infection status. Briefly, two parallel analyses were performed to segregate the influence of virus load or genotype on gene expression: first, high-responding HSV-1 infected *Rel*^*C307X*^ and *Rel*^+*/*+^ mice were compared directly, and second, all high-responding HSV-1 infected mice were compared to non-infected mice irrespective of genotype group. Only those genes that varied exclusively as function of genotype group (analysis 1), and that were not driven by HSV-1 infection (analysis 2), were retained (Supplementary Fig. 3b). 13 *Rel*^*C307X*^-dependent DEG were identified (Supplementary Fig. 3c), including galectin-family member *Lgals3* involved in innate and cellular immunity, CNS-tropic *Pgbd1,* and Serum/Glucocorticoid Regulated Kinase 1 (*Sgk1*) involved in cell survival^34^. Five long non-coding RNAs were also identified as *Rel*^*C307X*^-specific DEG that varied independently of the virus. Further investigation into these 13 genes may reveal a more direct involvement of c-Rel in their expression.

### High-responding *Rel*^*C307X*^ mice generate stronger IFN-stimulated and cell-mediated inflammatory responses to HSV-1 infection in the brainstem

**Figure 3:**
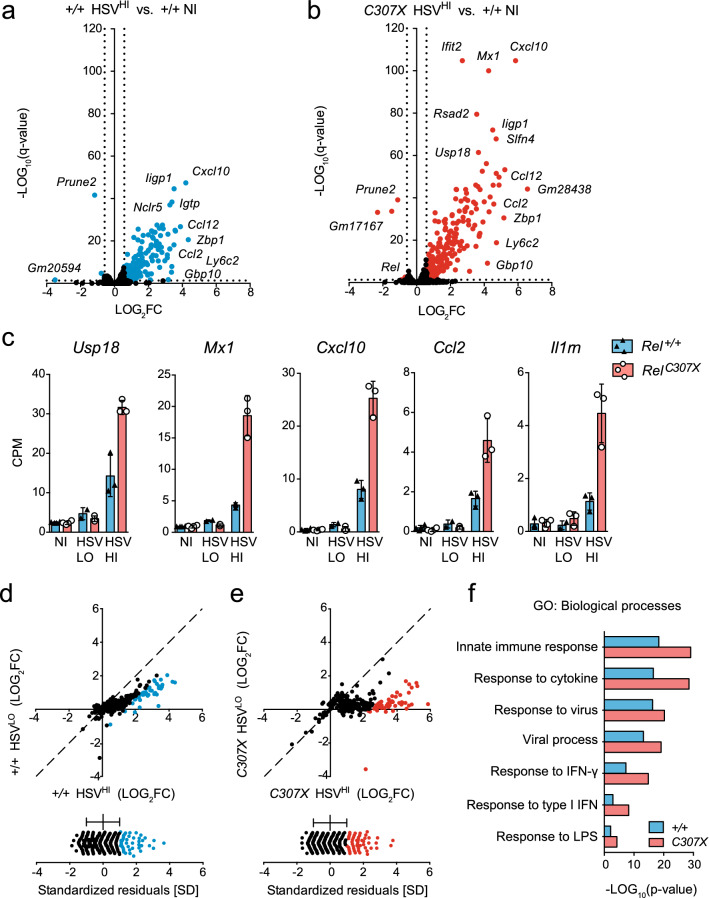
Elevated expression of defence response genes in high-responding *Rel*^*C307X*^ brainstems at day 5 post HSV-1 infection. Volcano plots of gene expression changes in (**a**) +*/*+ HSV^HI^ brainstems (*n* = 3 mice; 163 DEG) or (**b**) *C307X* HSV^HI^ brainstems (*n* = 3 mice; 256 DEG) compared to the +/+ NI group (*n* = 4 mice); DEG met a threshold of *q* < 0.05 (B-H-adjusted) and of ≥ 1.5-fold change in expression. (**c**) Expression of select DEG identified in (**a**,**b**) across all sample groups. (**d**,**e**) DEG for both +*/*+ HSV^HI^ and *C307X* HSV^HI^ high-responders are plotted against their corresponding fold change in each respective low-responding groups (upper panels). Upregulated genes for which standard residuals ≥ 1 (≥ 1 SD from the mean) are indicated in blue for +/+ groups, and in red for *C307X* groups (lower panels). (**f**) Representative gene ontology (GO) terms enriched (*p* < 0.05) in both +*/*+ and *C307X* gene groups whose standard residuals ≥ 1 as defined in (**e**,**f**). Data represent mean ± SD in (**b**,**c**). Nominal *p* values for enriched GO terms were assessed using DAVID in (**f**).

The impact of the mutation on host gene expression at day 5 post-HSV-1 infection was evaluated by independently comparing high-responding *Rel*^+*/*+^ and *Rel*^*C307X*^ infected groups separately to non-infected *Rel*^+*/*+^ controls. Here, 159 genes for *Rel*^+*/*+^ and 249 genes for *Rel*^*C307X*^ were upregulated by each group in response to infection (Fig. 3a,b). However, for many of these upregulated genes—like ISGs *Usp18* and *Mx1*, and secreted factors including *Cxcl10* and *Ccl2* chemokine genes and IL-1 receptor agonist *Il1rn*—their expression was markedly higher in *Rel*^*C307X*^ mice (Fig. 3c). Many other genes involved in IFN-I or IFN-II signaling were also generally upregulated in *Rel*^*C307X*^ mice, while IFN receptors were steadily expressed at all timepoints in the brainstem (Supplementary Fig. 4a,b). To confirm that the defence response as a whole was heightened in mutant animals, fold-wise gene expression was compared between respective low- and high-responding groups (Fig. 3d,e). Briefly, for either genotype group, genes expressed in high- and low-responders were plotted against each other, and standard residuals to the identity line were calculated. By considering only genes that skewed at greater than 1 standard deviation towards high-responders (with standard residuals ≥ 1), *Rel*^*C307X*^ brainstems were found to be more enriched for functional GO terms related to innate immune, cytokine (IFN-I and IFN-II) and virus responses (Fig. 3f). Thus, *Rel*^*C307X*^ mice respond to HSV-1 infection by expressing host defence and IFN-related genes at a much higher magnitude as early as day 5 p.i.

**Figure 4:**
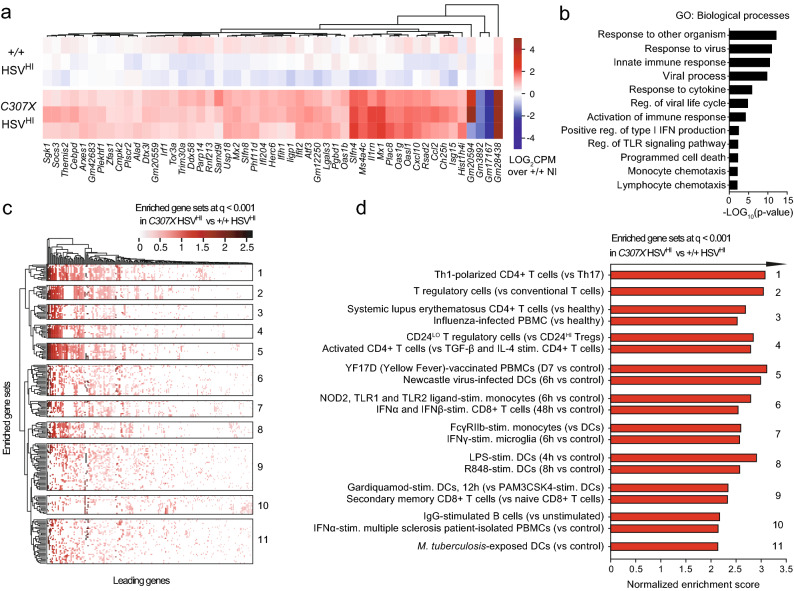
Dysregulated IFN and inflammatory pathways in high-responding HSV-1-infected *Rel*^*C307X*^ brainstems. (**a**) Heatmap of normalized gene expression of 51 DEG [≥ 1.5-fold change in expression, and *q* < 0.05 (BH-adjusted)] in *C307X* HSV^HI^ compared directly to +/+ HSV^HI^ (*n* = 3 mice per group) in the brainstem at day 5 post HSV-1 infection. (**b**) Gene ontology (GO) terms enriched (*p* < 0.05) across the 51 *C307X* HSV^HI^ DEG defined in (**a**). (**c**) Hierarchical clustering of leading genes that drive the enrichment of curated gene sets in *C307X* HSV^HI^ mice compared to +*/*+ HSV^HI^ mice using GSEA (at least *q* < 0.001, top *N* = 200 gene sets). (**d**) Normalized enrichment scores for select gene sets enriched in *C307X* HSV^HI^ mice (*q* < 0.001), representative of the 11 clusters defined in (**c**). Nominal *p* values for enriched GO terms were assessed using DAVID in (**b**), and *q* values (BH-adjusted) and normalized enrichment scores were assessed using GSEA in (**c**,**d**).

In a parallel analysis, 51 DEG were identified by directly comparing *Rel*^*C307X*^ high-responders to *Rel*^+*/*+^ high-responders (Fig. 4a). These *Rel*^*C307X*^-specific DEG were involved in IFN signaling, lymphoid and myeloid cell chemotaxis, and programmed cell death (Fig. 4b). Expanding beyond these 51 DEG using GSEA, *Rel*^*C307X*^-enriched gene sets were related to type 1 helper CD4^+^ T cells, regulatory T cells (Tregs), IgG-stimulated B cells, and Fc receptor-stimulated monocytes, in addition to various TLR-stimulations in DCs (Fig. 4c,d). The high-responding *Rel*^*C307X*^ gene expression landscape further overlapped with inflammatory gene sets derived from IFN-stimulated microglia, peripheral blood mononuclear cells and CD8^+^ T cells. Altogether, these enriched signatures reveal that the truncating mutation led to increases in IFN-stimulated responses and in the contribution of T and myeloid cell-mediated pathways to the *Rel*^*C307X*^ HSE susceptible phenotype.

### HSE onset is associated with elevated pathological infiltration of myeloid and T cells in the *Rel*^*C307X*^ brain

**Figure 5:**
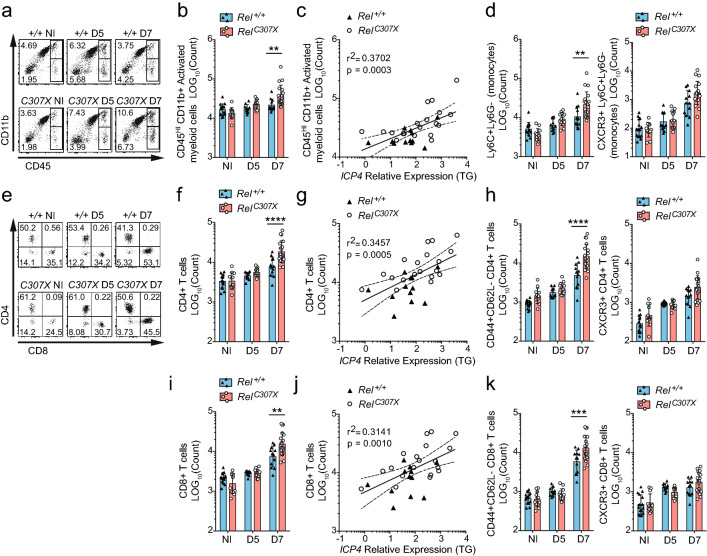
Myeloid and T cell infiltrations in the brain of high-responding *Rel*^*C307X*^ mice. Whole brain tissue was collected from HSV-1-infected *Rel*^+*/*+^ and *Rel*^*C307X*^ mice at day 5 p.i. (D5; *n* = 6F + 5M *Rel*^+*/*+^; *n* = 8F + 5M *Rel*^*C307X*^) and day 7 p.i. (D7; *n* = 7F + 5M *Rel*^+*/*+^; *n* = 11F + 6M *Rel*^*C307X*^), along with corresponding and non-infected controls (NI; *n* = 7F + 8M *Rel*^+*/*+^; *n* = 6F + 6M *Rel*^*C307X*^). (**a**) Flow cytometry plots of representative myeloid (CD45^HI^ CD11b^+^) and lymphoid (CD45^HI^ CD11b^−^) cell populations at NI, D5, and D7 timepoints, and indicating percentages of singlet and viable brain-isolated cells. (**b**) CD45^HI^ CD11b^+^ activated myeloid cells were enumerated, with (**c**) D7 cell counts plotted against viral *ICP4* relative expression in corresponding trigeminal ganglia (TG). (**d**) Ly6C^+^Ly6G^−^ (monocyte-like) activated myeloid cells were also quantified, as well as CXCR3^+^Ly6C^+^Ly6G^−^ cells. (**e**) Flow cytometry plots of representative CD4^+^ and CD8^+^ T cell populations at NI, D5 and D7 timepoints, and indicating percentages of singlet and viable CD45^HI^CD11b^−^CD3^+^NK1.1^−^ T cells. (**f**) CD4^+^ T cells were enumerated, and (**g**) D7 cell counts plotted against viral *ICP4* relative expression in corresponding TG. (**h**) CD44^+^CD62L^−^ and CXCR3^+^ CD4^+^ T cells were also quantified. (**i**) CD8^+^ T cells were enumerated, and (**j**) D7 cell counts plotted against viral *ICP4* relative expression in corresponding TG. (**k**) CD44^+^CD62L^−^ and CXCR3^+^ CD8^+^ T cells were also quantified. Full gating strategies are detailed in Supplementary Fig. 6. Experiments include male and female mice, and data represent mean ± SD. Statistical tests were performed in (**b**,**d**,**f**,**h**,**i**,**k**) using two-way ANOVA with Tukey’s multiple correction test, **p < 0.01, ***p < 0.001, ****p < 0.0001. For linear regressions in (**c**,**g**,**j**), 95% confidence intervals (dotted lines), r^2^, and *p* values are included to evaluate goodness-of-fit.

To confirm which resident or infiltrating cell types were populating the brain during HSV-1 infection, and therefore which cells were concurrent with altered gene expression, neuronal, glial and hematopoietic cells were quantified at various timepoints leading up to HSE symptom onset in *Rel*^*C307X*^ mice. As HSV-1 typically accedes to the hindbrain via the trigeminal ganglia (TG) in i.n. models of infection, we first examined the TG at day 4 p.i. (Supplementary Fig. 5a–e) and the brainstem at day 5 p.i, (Supplementary Fig. 5f–j) and found no major differences in the number of myeloid, CD4^+^ or CD8^+^ T cells between *Rel*^+*/*+^ and *Rel*^*C307X*^ groups. To better capture the global encephalitis response, whole brain tissue was processed at day 5 and later at day 7, near peak onset of symptoms in susceptible mutant mice. Here, at discrete timepoints, brain-resident neurons, oligodendrocytes and microglia, as well as infiltrating NK cells, B cells and neutrophils, remained unchanged in number across infection (Supplementary Fig. 5k–m). However by day 7 p.i., three major infiltrating populations were augmented in the *Rel*^*C307X*^ brain. First, increased numbers of CD45^HI^CD11b^+^ activated myeloid cells (Fig. 5a,b) were detected in the brain of *Rel*^*C307X*^ mice, and directly correlated with *ICP4* viral gene expression in adjacent TG at day 7 p.i. (Fig. 5c). Among these myeloid cells, the Ly6G^−^Ly6C^+^ monocyte subset was more elevated in *Rel*^*C307X*^ mice, and was also positive for CXCR3 expression, the cognate homing receptor for *Rel*^*C307X*^-upregulate chemokine CXCL10 (Fig. 5d). Second and third respectively, CD4^+^ and CD8^+^ T cells were also more numerous in *Rel*^*C307X*^ mice (Fig. 5e,f,i), and again in step with levels of HSV-1 replication (Fig. 5g,j). These T cells all expressed CXCR3 as the infection progressed, and exhibited an activated CD44^+^CD62L^−^ profile by day 7 p.i. (Fig. 5h,k). Of note, in wild-type mice at day 5 p.i., the expression of the full-length c-Rel protein was upregulated from steady-state levels predominantly in these activated myeloid cell, monocyte and T cell subsets, as well as in B cells (Supplementary Fig. 1f,d,h–k), suggesting that c-Rel is responding to infection in these key cell types. Overall, these associations between activated myeloid and T cell subsets with HSV-1 replication support a pathological role for these infiltrating cells in the development of HSE.

## Discussion

**Figure 6:**
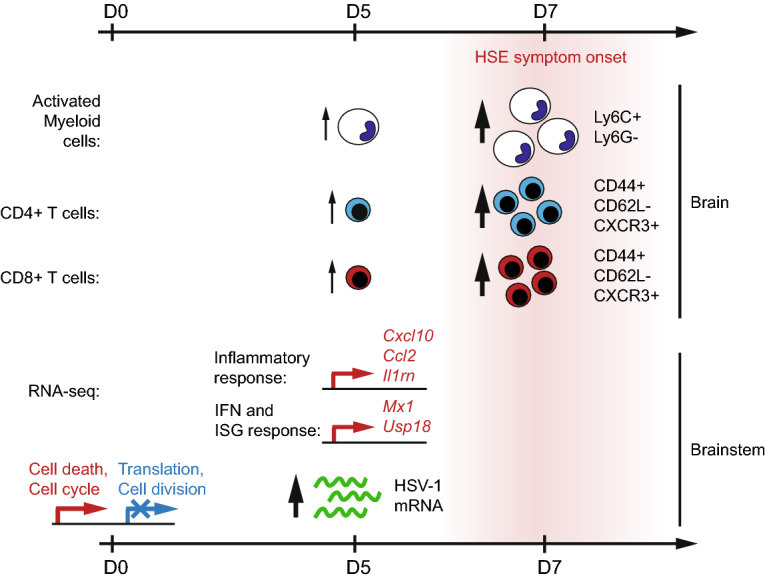
Progression of cellular infiltration and differential host and viral gene expression in high-responding HSV-1-infected *Rel*^*C307X*^ mice. Prior to day 0 (non-infected) and following intranasal HSV-1 infection (day 5), changes in the host and viral transcriptional landscape in high-responding *Rel*^*C307X*^ brainstems were measured by dual RNA-seq and are shown in the lower portion of the figure. Recruitment and infiltration of immune cells at day 5 and day 7 p.i. was assessed in the whole brain by flow cytometry, and illustrated in the upper portion of the figure. All increases or decreases are relative to *Rel*^+*/*+^ mice.

To our knowledge, this study is the first to employ host–pathogen gene expression profiling in an in vivo mouse model of HSE. Specifically, we have combined RNA-seq of HSV-1 infected brainstems together with flow cytometry of infiltrating and brain-resident cells to better understand the effect of the *Rel*^*C307X*^ mutation and contribution of c-Rel-dependent regulation to HSE susceptibility, modelled in Fig. 6. These strategies allowed for the detection of increased viral replication and host IFN-driven inflammatory responses as early as day 5 p.i., at least one full day before expected HSE symptom onset. High-responding *Rel*^*C307X*^ mice were also distinguished by elevated infiltration of myeloid and T cell subsets to the brain, involving these cells directly in the promotion of a pathological HSE response. Furthermore, the distribution of cellular infiltration and of viral and host transcripts in *Rel*^*C307X*^ brainstems closely recapitulated the expected survival outcome of these mice to intranasal HSV-1 infection: approximately 60% of *Rel*^*C307X*^ mice rapidly succumb to lethal HSE disease between days 6 to 9 p.i., while the remaining 40% of *Rel*^*C307X*^ mice and all wild-type littermates never develop HSE^29^. Consistent with these previously published data, we conclude that early c-Rel-dependent differences in host gene expression at day 5 p.i. are predictive of later HSE disease outcome.

The earliest effects of the *Rel*^*C307X*^ mutation on gene expression were detected at steady-state in non-infected adult brainstems, and were related to cell division, survival and homeostasis. The *Rel*^*C307X*^-specific enrichment of hypoxic and apoptotic responses was also notable; c-Rel has been implicated in pro-survival responses in neurons^35,36^, and as we have previously shown, *Rel*^*C307X*^ fibroblasts are more susceptible to caspase-3-dependent cell death following ex vivo HSV-1 infection^29^. Supporting a wider role for c-Rel in neuronal survival, *Rel*^*−/−*^ mice have been found to exhibit Parkinson’s disease-like symptoms and neurodegeneration at 18 months of age^37^. In the case of the *Rel*^*C307X*^ brainstem, it is tempting to speculate how subtle defects in c-Rel-dependent regulation of cell survival and homeostasis may be further exacerbated by age, injury or infection. Being that one of the final outcomes of HSE in susceptible *Rel*^*C307X*^ mice is elevated in vivo detection of cleaved caspase-3 in the infected brain^29^, these early basal differences in the *Rel*^*C307X*^ transcriptome merit further investigation, and may reflect an important contribution of the cell-resident compartment to HSV-1 susceptibility in our model.

In HSV-1 infected brainstems at day 5, the capture of viral mRNA sequences was pivotal to the identification of early high- and low-responding mice to infection. This subdivision of *Rel*^+*/*+^ and *Rel*^*C307X*^ mice into distinct response groups ensured that HSE-susceptible *Rel*^*C307X*^ mice could be analyzed apart from the other (approximately 40%) *Rel*^*C307X*^ mice that, due to the partial penetrance of the mutation, are expected to survive past the 14 day mark. Thus, compared to other response and genotype groups, high-responding *Rel*^*C307X*^ mice were found to have considerably more replicating virus while simultaneously upregulating IFN-dependent gene expression. These elevated ISG responses were surprising in HSE-susceptible *Rel*^*C307X*^ mice, and clearly distinguished our model from other HSE models where type I IFN is protective. For example, *Ifnar1*^*−/−*^ and *Stat1*^*−/−*^ knockout mice mount defective IFN-stimulated responses and succumb early to HSV-1-induced neuroinflammation^30,38^. The *Rel*^*C307X*^ model also deviated from TLR3- and NF-κB (NEMO)-related inborn defects in IFN that underlie cases of human HSE^12,39,40^. Instead, the *Rel*^*C307X*^ transcriptional profile recalled the excess ISG responses that typify human type I interferonopathies^41^ and that have also been reported in human inflammatory diseases like systemic lupus erythematosus (SLE) and rheumatoid arthritis (RA), or during infectious tuberculosis^42,43^. Itself, IFN expression can be toxic to neurons and exacerbate neurodegeneration following tissue injury^44,45^. While we did not previously observe a *Rel*^*C307X*^-specific defect in *Ifnb* expression ex vivo HSV-1-infected primary fibroblasts and macrophages^29^, a potential role for c-Rel in the regulation of IFN-I expression warrants further investigation in vivo and across multiple cell types. Overall, the *Rel*^*C307X*^ model demonstrates how the coexistence of increased ISG responses with increased viral replication can be pathological, and illustrates well the need for a balanced IFN-dependent response in achieving protection against HSE.

A further subset of inflammatory genes that were induced in *Rel*^*C307X*^ brainstems above wild-type levels included interleukin-1 receptor antagonist-encoding *Il1rn*, and chemokine-encoding genes *Ccl2* and *Cxcl10*. At day 5 p.i., these DEG were detected prior to infiltration of immune cells to the brainstem, suggesting that they may have been expressed by microglia or other brain-resident cells^19,46^. Polymorphisms in human *IL1RN* have been associated with in vivo control of Epstein–Barr viral load, and with inflammatory bowel and skin disorders that have also been associated with the *REL* locus^47–49^. On the other hand, *Cxcl10* is notable for being induced and regulated by c-Rel in T cells^50^. Finally, the production of both CCL2 and CXCL10 chemokines in the HSV-1-infected brain are responsible for recruiting CCR2^+^ and CXCR3^+^ monocytes respectively, as well as CXCR3^+^ T cells, which typically exacerbate HSE pathology^24,26,27^. In the case of high-responding *Rel*^*C307X*^ mice, the significance of increased chemokine and inflammatory gene expression was two-fold. First, the detection inflammatory gene signatures at day 5 p.i. was an important early marker for the later surge in T helper type 1 (Th1) cytokine and chemokine production previously observed in the hindbrains of HSE-symptomatic *Rel*^*C307X*^ mice from day 7 p.i. onwards^29^. Second, the early induction of *Ccl2* and *Cxcl10* in the brainstem was likely related to the observed pathological recruitment of myeloid and CXCR3^+^ T cells at day 7 p.i. in the *Rel*^*C307X*^ brain. These infiltrating cells, particularly Ly6C^+^ monocytes, CD4^+^ and CD8^+^ T cells, all upregulated c-Rel protein levels in wild-type mice in response to infection at day 5 p.i., suggesting that c-Rel participates in their transcriptional response and may modulate the function of these cells. Notably, *Rel*^*−/−*^ mice do not exhibit defects in antiviral T cell responses to influenza infection^51^, and neither were peripheral CD4^+^ or CD8^+^ T cells impaired in their ability to produce IFN-γ at day 5 post-HSV-1 infection in *Rel*^*C307X*^ mice^29^. Instead, we propose that the *Rel*^*C307X*^ mutation disrupts the normal regulation of T cells in their capacity to mediate inflammation, resulting in early increases in inflammatory gene expression and culminating in the late and excessive infiltration of activated T and myeloid cells to the brain during lethal HSE.

The *Rel*^*C307X*^ mouse is also set apart from most other HSE models by the partial penetrance of the C307X mutation, which resulted in aberrant IFN-driven inflammatory responses and lethal HSE onset in approximately 60% of *Rel*^*C307X*^ mice. Partial susceptibility to HSE is also observed in full *Rel*^*−/−*^ knockout and compound heterozygous *Rel*^*C307X/−*^ mice^29^. Thus, the incomplete penetrance of the mutation is perhaps linked to the nature of c-Rel as a transcription factor, and stemming from NF-κB subunit dynamics which are complex, tightly regulated, and context-specific^52,53^. The formation and configuration of active NF-κB dimers, as the most proximal event to nuclear entry and DNA binding, is likely to influence the target gene expression. While C307X c-Rel homodimers may be unable to directly modulate transcription due to a premature truncation of the transactivation domain, the formation of heterodimers between C307X c-Rel and intact p65 or p50 subunits may still regulate transcription^54^. As a notable parallel, the transcription factor IRF3 can also homodimerize, or heterodimerize with IRF7 to modulate inflammatory gene transcription; the partial susceptibility of *Irf3*^*−/−*^ mice to HSE, but complete susceptibility of *Irf3*^*−/−*^*Irf7*^*−/−*^ double knockouts, supports the concept that the structure of dimeric transcription factors can influence HSE development in mice^55^. In the *Rel*^*C307X*^ model, further investigation into the cellular and tissue contexts that involve c-Rel-containing dimers, and how the C307X c-Rel subunit may interact with other NF-κB subunits or alter their availability or stability in complex, might improve our understanding of the *Rel*^*C307X*^ mutation’s penetrance. Where our tandem use of dual RNA-seq and flow cytometry has clarified the involvement of several different brain-resident and infiltrating cell types in the partial penetrant *Rel*^*C307X*^ phenotype, a single cell approach would also be well-suited to determine if *Rel*^*C307X*^-dependent defects are triggered uniquely in infected cells by viral replication, or occur in multiple cell types driven by the inflammatory milieu. Thus, infiltrating T and myeloid cells and brain-resident cells, isolated from the in vivo HSV-1-infected CNS at early timepoints, would be worth examining using single cell RNA-seq. This method would also allow the contribution of T cell subsets present in low abundance in the hindbrain to be assessed, including T regulatory (Tregs) and T helper type 17 (Th17) cells that both depend directly on c-Rel for their development and maturation, with Tregs known to be depleted in the *Rel*^*C307X*^ periphery^29,56,57^.

In summary, the *Rel*^*C307X*^ HSV-1 infection model captured an altogether different aspect of HSE disease compared to cases of inherited type I IFN/TLR3 axis deficiencies. *Rel*^*C307X*^ mice were instead defined by excess IFN-stimulated and neuroinflammatory responses, in turn attracting pathological T and myeloid cells to the brainstem whose resident cells, showing early signs of dysregulated cell cycle and survival, ultimately failed to tolerate inflammation and to control viral replication. In the wider context of human disease, the human *REL* gene has been associated with multiple inflammatory diseases^58,59^, and has recently been implicated in broad susceptibility to chronic human herpesvirus-5, *Salmonella* and *Cryptosporidium* infections in a c-Rel-deficient patient^60^. These studies, together with our findings that c-Rel promotes cell-mediated host defence against HSV-1 infection in mice, further support that c-Rel regulatory networks are at the intersection of host defence and inflammation. Thus, the *Rel*^*C307X*^ mouse constitutes an experimental model where c-Rel or its upstream regulators may potentially be targeted to reduce inflammation and long-term sequelae in the HSV-1-infected brain.

## Materials and methods

### Mice and virus infection

*Rel*^*C307X*^ mice (MGI:6287253 or *Rel*^*Coby*^ allele on the Mouse Genome Informatics (MGI) database, http://www.informatics.jax.org/) were discovered in an *N*-ethyl-*N*-nitrosourea mutagenesis screen as previously described^29^. Inbred *Rel*^*C307X*^ mice were backcrossed at least 4 times to the C57BL/6 background (The Jackson Laboratory), and are maintained in a breeding colony including littermate homozygous mutant *Rel*^*C307X*^ and homozygous wild-type *Rel*^+*/*+^ mice at McGill University. *Rel*^*C307X*^ mice have not demonstrated sex-specific differences affecting HSE susceptibility, viral replication or host responses^29^. Female mice were selected for RNA-seq experiments to avoid introducing sex as a covariate when comparing groups for differential gene expression, whereas both male and female mice were included in flow cytometry experiments.

For HSV-1 infections, as previously described in^29^, 7-week-old or older mice were first anaesthetized via intraperitoneal (i.p.) ketamine and xylazine injection, and infected with 5 × 10^4^ PFU of HSV-1 strain 17 per 20 g body weight via intranasal (i.n.) inoculation. The inoculum was delivered in 10 μl of sterile PBS per 20 g body weight in the left nostril with a micropipette, and allowed to be completely inhaled. Infected animals were weighed daily, monitored at least daily, and up to three times a day during the peak of infection (days 6–10 p.i.). Mice were euthanized upon demonstration of HSE-like symptoms (hunched posture, reduced mobility, neurologic symptoms) or of 15% loss of initial pre-infection body weight, or upon reaching experimental endpoints at day 5 or 7 p.i.

### Tissue collection and RNA preparation

At clinical or experimental endpoint, mice were euthanized and quickly perfused transcardially through the left ventricle with 10 ml cold PBS. As specified in each figure legend, either the brainstem, both TG (as described in^61^), or the whole brain (including the olfactory bulbs, cerebrum, cerebellum and brainstem) were excised and collected in either 5 ml Hibernate-A medium (ThermoFisher Scientific) at 4 °C for downstream flow cytometry, or snap-frozen in liquid nitrogen and conserved at − 80 °C for downstream RNA extraction. Snap-frozen brainstem or TG samples were transferred to 1 ml TRIzol reagent (Invitrogen) and homogenized at speed 6000 for 30 s with a MagNA Lyser Instrument (Roche). Total RNA was extracted as per the manufacturer’s standard protocol. RNA samples were further cleaned-up using the RNeasy Mini Kit (Qiagen #74104) and following DNase I treatment, as per the manufacturer’s standard protocol. Purified RNA was reverse transcribed into cDNA and real-time quantitative PCR was performed as previously described^29^, using the following primer pairs: *ICP4* (*RS1*) (forward 5′ CGACACGGATCCACGACCC 3′, reverse 5′ GATCCCCCTCCCGCGCTTCGTCCG 3′) and *Hprt* (forward 5′ CAGGCCAG-ACTTTGTTGGAT 3′, reverse 5′ TGGCGCTCATCTTAGGCTTT 3′).

### Dual RNA-seq and differential gene expression analysis

RNA-seq was performed as previously described^62^. Briefly, total RNA was purified from whole brainstem samples and assayed for RNA integrity using a Bioanalyzer RNA Pico kit (Agilent). cDNA libraries were generated after rRNA depletion with the KAPA Stranded RNA-Seq kit (Roche). Paired-end 50 bp read sequencing was performed using an Illumina HiSeq 2500 sequencer. Low-quality bases (Phred < 33) and adaptor sequences were removed with the Trimmomatic v.0.36 tool^63^ using the following arguments: ILLUMINACLIP:TruSeq3-PE.fa:2:30:10 HEADCROP:4 LEADING:5 TRAILING:3 SLIDINGWINDOW:4:20 MINLEN:36. All trimmed reads were first aligned to the mouse GRCm38 (mm10) reference genome using TopHat2 v2.1.1 with Bowtie2 v2.3.1 algorithms^64,65^, and were quantified per gene by counting the number of strand-specific reads aligning to gene exon features using the featureCounts tool (Subread package v1.5.2^66^). In parallel, using HISAT2 v2.1.0^67^, all trimmed reads were also aligned to an indexed HSV-1 strain 17 reference genome^68^ (GenBank: JN555585.1). Viral reads were quantified per viral gene by counting the number of strand-specific reads aligning to gene and/or exon features using featureCounts (Supplementary Data File 1). Here, in addition to default arguments, the “M” and “primary” arguments were used to only count multiple-mapping reads once at their primary alignment site, and avoid to counting twice over genes that are naturally duplicated in the HSV-1 genome (*RS1*, *RL1*, *RL2*, *LAT*).

Raw read counts libraries for host gene were first filtered to remove residual rRNA reads, and to only keep genes expressed above 3 counts per million host reads (CPM) in at least 3 samples, for a total of 16,279 expressed host genes. For viral reads, all expressed viral genes were retained and were similarly normalized per million host reads. Filtered host count libraries were normalized with the TMM method and differential host gene expression was assessed pairwise between sample groups using the edgeR Bioconductor package^69^. Genes whose expression was ≥ ± 1.5 fold change between groups, and that met a threshold of *q* < 0.05 (Benjamini–Hochberg (BH)-adjusted p value), were considered statistically significant (Supplementary Data File 2). Gene expression heatmaps of CPM values per sample, normalized to the average CPM values across a control group specified in each figure, were generated using the “gplots” package in R and clustered gene-wise using a Euclidean distance measure.

### Gene ontology term and gene set enrichment analyses

Gene ontology (GO) term enrichment analysis for biological processes (BP4) was performed on differentially expressed genes identified by RNA-seq between *Rel*^+*/*+^ and *Rel*^*C307X*^ groups at select time points of infection using the DAVID v.6.8 online database^70^. Enriched GO terms that met a nominal *p*-value cut-off of 0.05 were considered significant (Supplementary Data File 3). To identify cellular or immune pathways that were present in either *Rel*^+*/*+^ and *Rel*^*C307X*^ mice gene expression profiles (including all 16,279 expressed genes), gene set enrichment analysis was performed using GSEA^71^ to detect enrichment of gene sets publicly listed in the MSigDB v6.2 collection, specifically among *N* = 3406 Curated Gene Sets (C2, including chemical and genetic perturbations, canonical pathways (BIOCARTA, KEGG, PID or REACTOME) or *N* = 4872 Immunological Signatures (C7). Gene sets that met a Benjamini–Hochberg (BH)-adjusted *p* value cut-off of at least *q* < 0.05 were considered significantly enriched in each condition. Furthermore, enriched gene sets were clustered according to shared leading edge genes (using Manhattan distance) to group similar or redundant signatures together, and better resolve general themes specific to *Rel*^*C307X*^ or *Rel*^+*/*+^ mice. For these leading edge analyses, only those genes that were ranked prior or at the position of the gene with the maximum enrichment score in any given gene set (the leading edge), and that were represented in at least 5% of all enriched gene sets, were included for hierarchical clustering (Supplementary Data File 4).

### Flow cytometry

Adapted from^72^ with modifications, freshly excised HSV-1 infected whole brain, brainstem or TG tissue were collected in 5 ml Hibernate A medium at 4 °C and were processed into single cell suspensions and stained for flow cytometry. Briefly, tissues were minced and digested in 1 ml 1X HBSS containing 510 U/ml collagenase II (Worthington Biochemical Corp.) and 28 U/ml DNase1 (Roche) for 15 min at 37 °C. 9 ml 1X HBSS 2 mM EDTA were added to digested samples, and the remaining tissue was gently homogenized through a 140 μm mesh in a cell dissociation sieve (Sigma #CD1) to generate a single cell suspension. Cells were pelleted, resuspended in a 37% Percoll™ solution, and centrifuged at 500×*g* for 20 min with no break. The top-floating myelin/debris layer and the supernatant was removed by aspiration, and the cell pellet (containing hematopoietic immune cells, neurons, oligodendrocytes, microglia, and any remaining red blood cells or debris) was washed twice and resuspended in 1X PBS 2% FBS 2 mMol EDTA to accommodate two antibody staining panels. Cells were treated with anti-CD16/CD32 to block non-specific binding to Fc receptors (eBioscience #16-0161), and were stained with extracellular antibodies overnight at 4 °C. The following fluorochrome-conjugated antibodies (clone, working dilution) were used from eBioscience (Invitrogen): B220 APC (RA3-6B2, 1:100), CD11b eFluor450 (M1/70, 1:50), CD44 FITC or PE (IM7, 1:100), CD45 PerCP-cy5.5 (30-F11, 1:200), CD62L FITC or PE (MEL-14, 1:100), CXCR3 PE-cy7 (CXCR3-173, 1:100), NK1.1 PE-cy7 (PK136, 1:100); from BioLegend: CD3 Brilliant Violet 605 (17A2, 1:100), CD4 Brilliant Violet 510 (GK1.5, 1:100), CD8a eFluor450 or Brilliant Violet 785 (53-6.7, 1:100), I-A/I-E (MHC-II) AlexaFluor700 (M5/114.15.2, 1:300), Ly6G Brilliant Violet 711 (1A8, 1:500), Ly6C Brilliant Violet 785 (HK1.4, 1:2000); and from Miltenyi Biotec: O4 APC (O4, 1:50). Cells were later stained with Fixable Viability Dye (eFluor780-conjugated or eFluor506-conjugated, eBioscience #65-0865 or #65-0866) to mark dead cells. Next, cells were fixed and permeabilized with the Foxp3/Transcription Factor Staining Buffer Kit (eBioscience #00-5523-00) as per the manufacturer’s instructions, and stained with the following intracellular antibodies (clone, working dilution, supplier): c-Rel PE (1RELAH5, 1:100, eBioscience) and NeuN AlexaFluor488 (EPR12763, 1:25, Abcam). 5000 counting beads (Spherotech #ACBP-50-10) were added to each sample prior to acquisition on a BD LSRFortessa cytometer. Total cell counts for each cell population were normalized to the total number of beads per sample and to the total number of singlet viable cells acquired in both staining panels. Cell populations were gated as described in Supplementary Fig. 6 using FlowJo v. 10.1 software.

### Statistics

Statistical tests were performed using the statistical package of GraphPad Prism v. 6 software, as detailed in each figure legend. Two-way ANOVA were performed with Tukey’s multiple correction test between pairwise groups, where adjusted *p* values < 0.05 were considered significant (*p < 0.05, **p < 0.01, ***p < 0.001, ****p < 0.0001).

### Ethics statement

All animals used in this study were housed and maintained at McGill University. All experiments were performed under the guidelines and recommendations of the Canadian Council on Animal Care (https://ccac.ca/en/standards/guidelines/). The animal use protocol was approved by the McGill University Animal Care Committee (protocol number 4792). The study was carried out in compliance with the ARRIVE guidelines (https://arriveguidelines.org).

## Supplementary Information

Supplementary Information 1.

Supplementary Information 2.

Supplementary Information 3.

Supplementary Information 4.

Supplementary Information 5.

## Data Availability

RNA-seq data are available in the National Center for Biotechnology Information (NCBI) Gene Expression Omnibus (GEO) database under the following accession number: GSE168799.
