# Supplementary material for: The c-Rel transcription factor limits early interferon and neuroinflammatory responses to prevent herpes simplex encephalitis onset in mice
Source: Sci Rep. 2021 Oct 27;11:21171. doi: 10.1038/s41598-021-00391-7 (PMC8551191; doi:10.1038/s41598-021-00391-7)
Supplement: Supplementary file 5 — Supplementary Information 5. [file 41598_2021_391_MOESM5_ESM.pdf]

Supplementary Information

The c-Rel transcription factor limits early interferon and neuroinflammatory responses to prevent herpes simplex encephalitis onset in mice

Mathieu Mancini,[1,2] Benoît Charbonneau,[1,2] David Langlais,[1,2,3] and Silvia M. Vidal[1, 2,4,*]

[1]Department of Human Genetics, McGill University, Montreal, QC, Canada

[2]McGill University Research Centre on Complex Traits, McGill University, Montreal, QC, Canada

[3]McGill University Genome Centre, Montreal, QC, Canada

[4]Department of Medicine, McGill University, Montreal, QC, Canada

*Corresponding author: Silvia M. Vidal, Email silvia.vidal@mcgill.ca

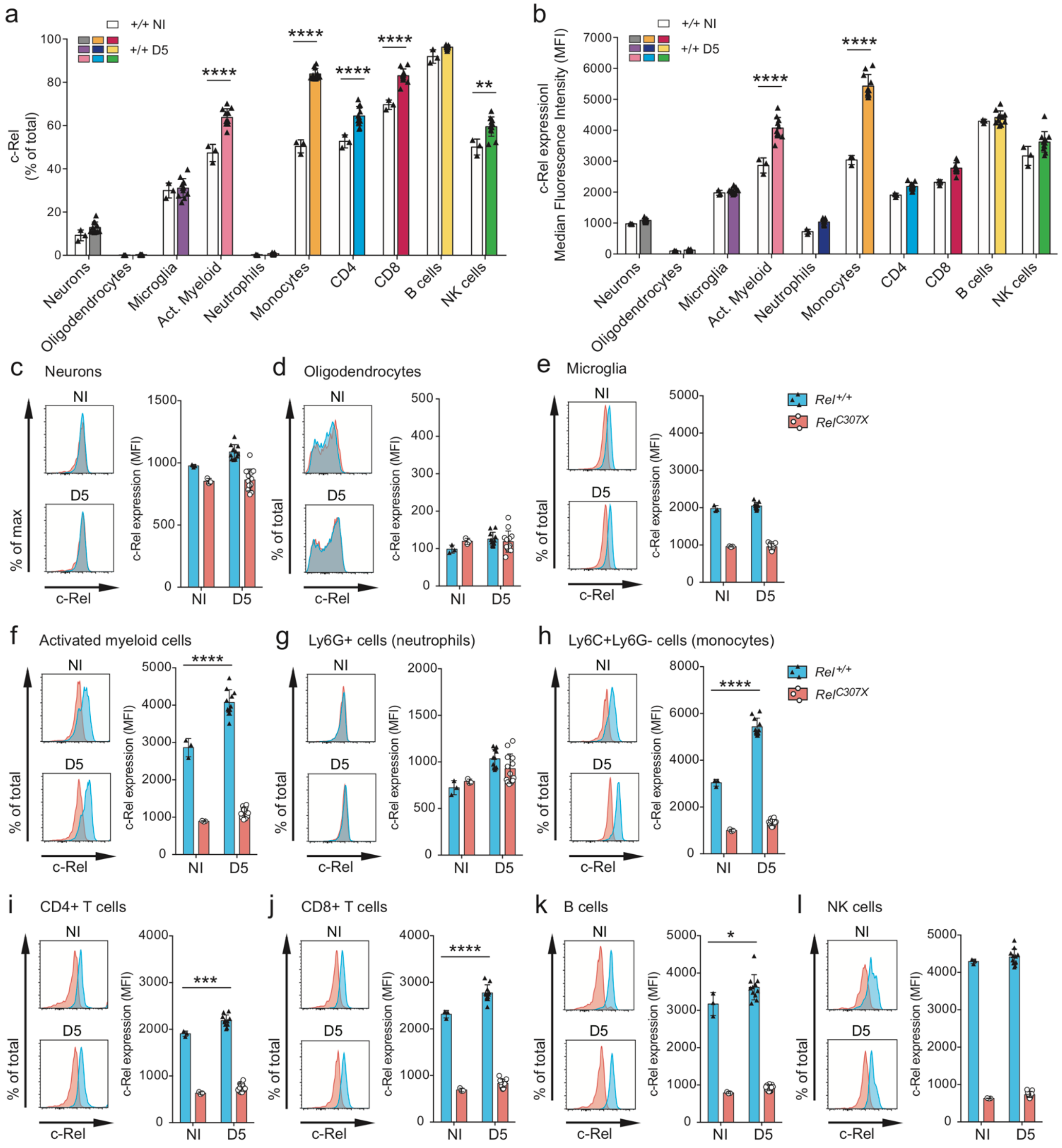

Supplementary Figure 1. c-Rel expression in resident and infiltrating cells of the brain.

Supplementary Figure 1. c-Rel expression in resident and infiltrating cells of the brain. Expression of c-Rel as a **(a)** percent of total cells and **(b)** median fluorescence intensity (MFI) for major resident and infiltrating cell populations in the wild-type $Rel^{+/+}$ whole brain at steady-state (NI in white; *n* = 3F mice) or at day 5 post HSV-1 infection (D5 in colour; *n* = 6F + 5M mice). Representative histograms depicting c-Rel expression as fluorescence intensity or quantified by MFI for NI (*n* = 3F $Rel^{+/+}$; *n* = 3F Rel^{C307X}) and D5-infected (*n* = 6F + 5M $Rel^{+/+}$; *n* = 8F + 5M Rel^{C307X}) groups in **(c)** neurons, **(d)** oligodendrocytes, **(e)** microglia, **(f)** Activated myeloid cells, **(g)** Ly6G^{+} neutrophil-like and **(h)** Ly6C^{+}Ly6G^{-} monocyte-like activated myeloid cells, **(i)** CD4^{+} T cells, **(j)** CD8^{+} T cells, **(k)** B cells, and finally **(l)** NK cells. Full gating strategies are detailed in Supplementary Fig. 6. Experiments include male (M) and female (F) mice, and data represent mean ± SD. Statistical tests were performed in **(a-l)** using two-way ANOVA with Tukey's multiple correction test, *$p < 0.05$, **$p < 0.01$, ***$p < 0.001$, ****$p < 0.0001$.

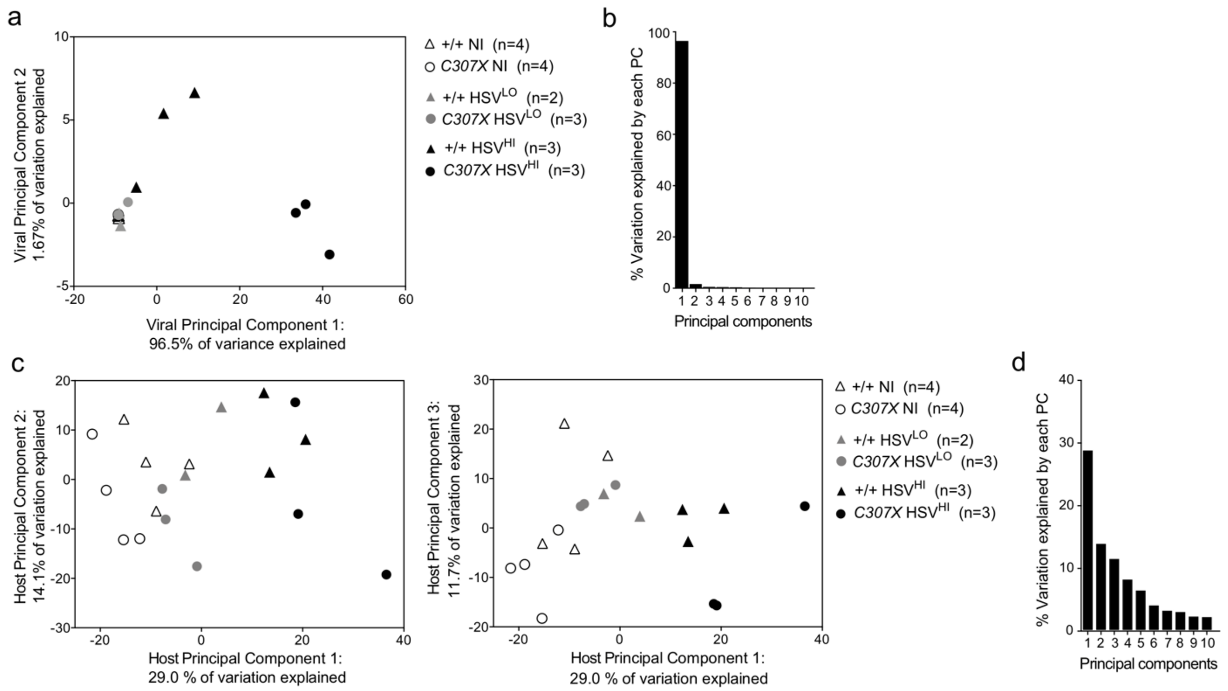

Supplementary Figure 2. Dimension reduction analyses of viral and host gene expression.

Supplementary Figure 2. Dimension reduction analyses of viral and host gene expression. Dual RNA sequencing was performed on brainstems collected from 19 HSV-1-infected mice, here labeled and divided into 6 groups as defined in Fig. 1, first by genotype, and further by either non-infected (NI) mice, or low-responders at day 5 p.i. with HSV-1 (HSV^{LO}), or high-responders at day 5 p.i. with HSV-1 (HSV^{HI}). **(a)** Principal component analysis (PCA) performed for all 19 samples across all 75 viral transcripts, with PC1 plotted against PC2. **(b)** Percentage of total variation explained by the first viral 10 PC in **(a)**. **(c)** PCA performed for all 19 samples across all 16,279 host genes expressed at > 3 CPM in at least 3 samples, with PC1 plotted against PC2 (left panel), and with PC1 plotted against PC3 (right panel). **(d)** Percentage of total variation explained by the first ten host PC in **(c)**.

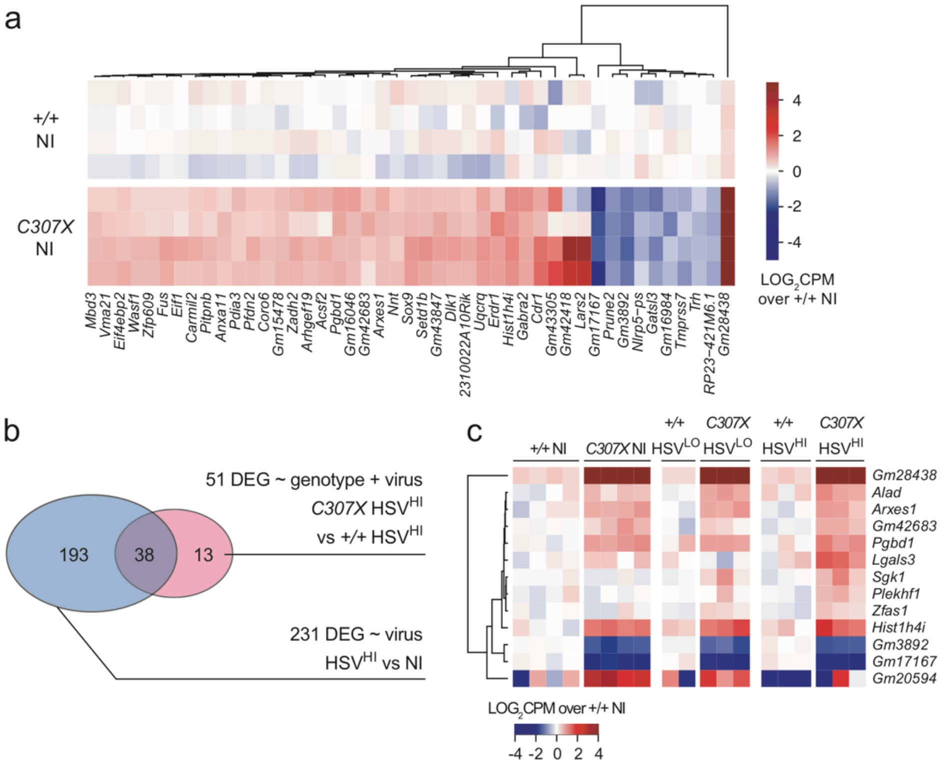

Supplementary Figure 3. ***Rel*^{C307X} genotype-specific differentially expressed genes in non-infected brainstems and across HSV-1-infected groups.**

Supplementary Figure 3. ***Rel^{C307X}*** **genotype-specific differentially expressed genes in non-infected brainstems and across HSV-1-infected groups.**

(a) Heatmap of normalized gene expression of 45 DEG [≥1.5-fold change in expression, and $q < 0.05$ (BH-adjusted)] in *C307X* NI compared directly to +/+ NI ($n = 4$ mice per group). **(b)** 231 total virus-driven DEG [≥1.5-fold change in expression, and $q < 0.05$ (BH-adjusted)] were identified by comparing all high-responding HSV^{HI} mice ($n = 6$) against all NI mice ($n = 8$), irrespective of genotype. Of the 51 DEG identified between *C307X* HSV^{HI} and +/+ HSV^{HI} mice in Fig. 4a, 13 DEG varied only due to the contribution of the *Rel^{C307X}* genotype. **(c)** Heatmap of normalized expression of these 13 *Rel^{C307X}*-specific DEG across all sample groups.

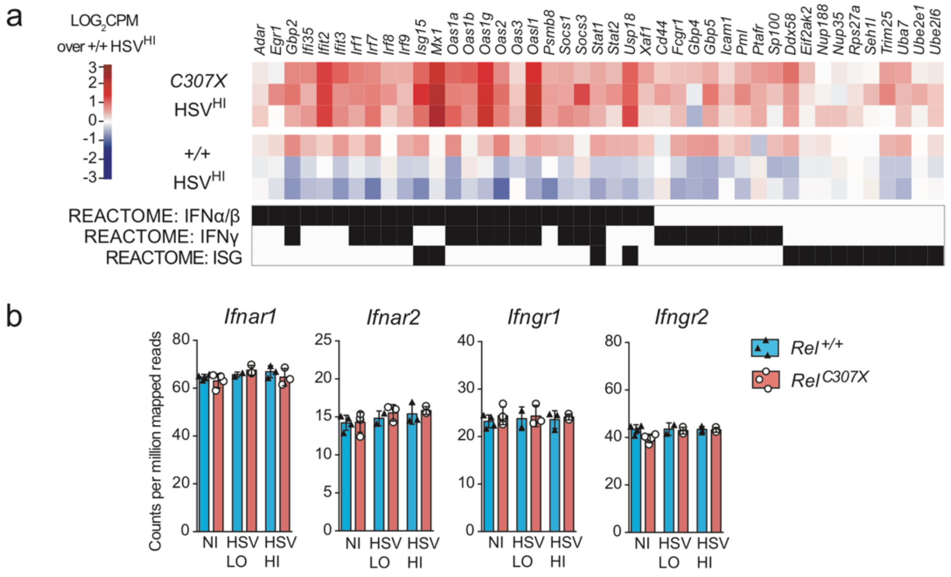

Supplementary Figure 4. Expression of IFN-related genes in HSV-1-infected brainstems.

Supplementary Figure 4. Expression of IFN-related genes in HSV-1-infected brainstems.
(a) Heatmap of normalized expression of select IFN-related genes defined in the REACTOME pathways "Interferon alpha/beta signaling", "Interferon gamma signaling", and "Antiviral mechanism by IFN-stimulated genes" in *C307X* HSV^{HI} brainstems compared to +/+ HSV^{HI} brainstems. **(b)** Expression of IFN-I and IFN-II receptor genes in the brainstem across all sample groups.

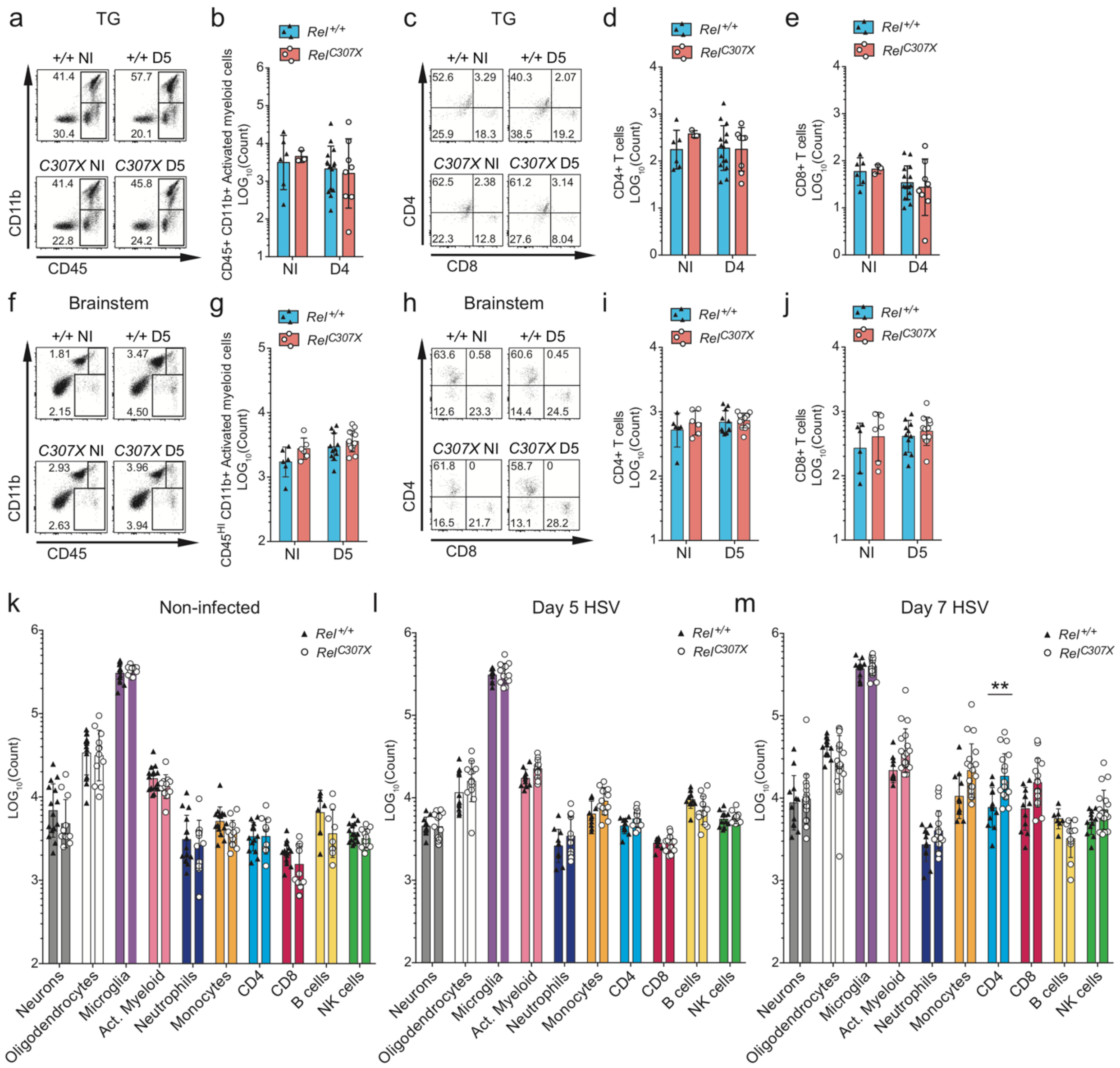

Supplementary Figure 5. Flow cytometry analysis of CNS-resident and infiltrating cells.

Supplementary Figure 5. Flow cytometry analysis of CNS-resident and infiltrating cells. **(a)** TG were collected from HSV-1-infected $Rel^{+/+}$ and Rel^{C307X} mice at day 4 (D4; n = 10F + 5M $Rel^{+/+}$; n = 5F + 3M Rel^{C307X}) along with corresponding and non-infected controls (NI; n = 4F + 2M $Rel^{+/+}$; n = 2F + 1M Rel^{C307X}). Flow cytometry plots of representative myeloid ($CD45^+CD11b^+$) and lymphoid ($CD45^+CD11b^-$) cell populations indicate percentages of singlet and viable TG-isolated cells, and $CD45^+CD11b^+$ activated myeloid cells are quantified in **(b)**. **(c)** In the TG, flow cytometry plots of representative $CD4^+$ and $CD8^+$ T cell populations indicate percentages of $CD45^+CD11b^-CD3^+NK1.1^-$ T cells, and **(d)** $CD4^+$ and **(e)** $CD8^+$ T cells are quantified. **(f)** Brainstems were collected from HSV-1-infected $Rel^{+/+}$ and Rel^{C307X} mice at day 5 (D5; n = 11F $Rel^{+/+}$; n = 13F Rel^{C307X}) along with corresponding and non-infected controls (NI; n = 6F $Rel^{+/+}$; n = 6F Rel^{C307X}). Flow cytometry plots of representative myeloid ($CD45^{HI}CD11b^+$) and lymphoid ($CD45^+CD11b^-$) cell populations indicate percentages of singlet and viable brainstem-isolated cells, and $CD45^{HI}$ $CD11b^+$ activated myeloid cells are quantified in **(g)**. **(h)** In the brainstem, flow cytometry plots of representative $CD4^+$ and $CD8^+$ T cell populations indicate percentages of $CD45^+CD11b^-CD3^+NK1.1^-$ T cells, and **(i)** $CD4^+$ and **(j)** $CD8^+$ T cells are quantified. In whole brain samples corresponding with Fig. 5, all myeloid, lymphoid and brain-resident cell populations are enumerated in $Rel^{+/+}$ and Rel^{C307X} mice by timepoint, namely NI **(k)**, day 5 post-HSV-1 infection **(l)**, and day 7 post-HSV-1 infection **(m)**. Full gating strategies are detailed in Supplementary Fig. 6. Experiments include male (M) and female (F) mice, and data represent mean ± SD. Statistical tests were performed in **(b, d, e, g, i, j-m)** using two-way ANOVA with Tukey's multiple correction test, $^{**}p < 0.01$.

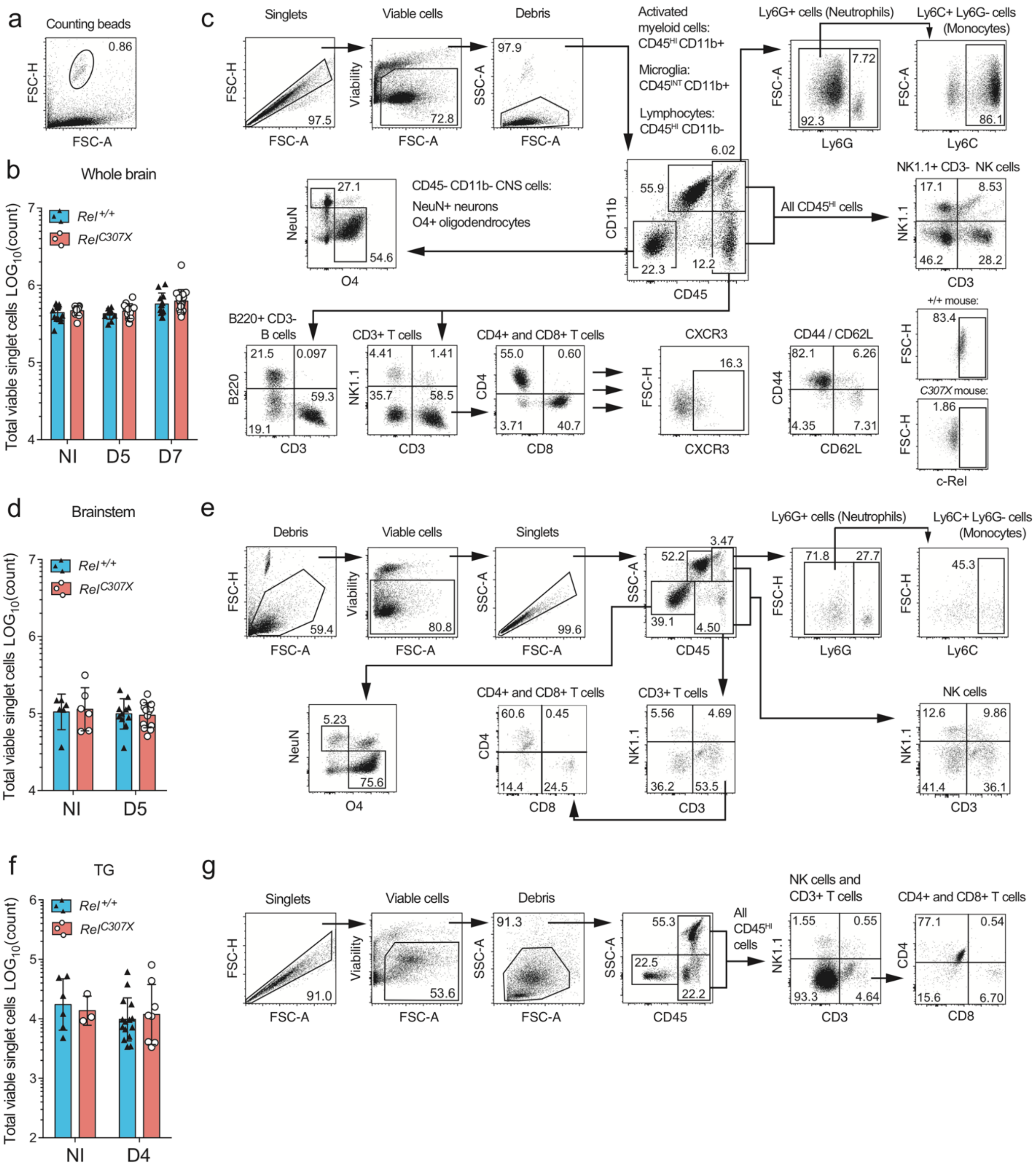

Supplementary Figure 6. Gating strategies for flow cytometry in the brain.

Supplementary Figure 6. Gating strategies for flow cytometry in the brain.

Whole brain, brainstem and trigeminal ganglia (TG) tissues were processed and single cell suspensions isolated and stained for flow cytometry. **(a)** Counting beads (5000 per sample) added to each sample immediately prior to acquisition. **(b)** Total singlet, viable, and debris-excluded cells quantified per sample in the whole brain. **(c)** Representative gating strategy for cells acquired in day 7 HSV-infected whole brain samples. Cells were first gated on singlet and viable populations, and debris gated out, followed by the separation of key brain-resident and infiltrating populations using CD11b and CD45 markers. From $CD45^{-}CD11b^{-}$ resident cells, neurons ($NeuN^{+}$) and oligodendrocytes ($O4^{+}$) were identified, while resident microglia are $CD45^{INT}CD11b^{+}$. Activated myeloid cells ($CD45^{HI}CD11b^{+}$) may include both inflammatory infiltrating myeloid cells or activated microglia, and were further separated into $Ly6G^{+}$ (neutrophil-like) or $Ly6G^{-}Ly6C^{+}$ (monocyte-like). $NK1.1^{+}CD3^{-}$ NK cells, which may temporarily express $CD11b^{+}$ in their penultimate stage of development, were gated from all $CD45^{HI}$ cells. Lymphocytes ($CD45^{HI}CD11b^{-}$) were gated into $B220^{+}CD3^{-}$ B cells and $CD3^{+}NK1.1^{-}$ T cells, and these T cells further by $CD4^{+}$ and $CD8^{+}$ expression. Further endophenotypes included $CXCR3^{+}$, $CD44^{+}$ or $CD62^{+}$ expressing subsets. Finally, c-Rel expression was quantified in wild-type $Rel^{+/+}$ mice, with Rel^{C307X} mice serving as a biological negative control to set the c-Rel positive gate, owing to the C-terminal-specific anti-c-Rel antibody not recognizing the truncated C307X c-Rel protein. **(d)** Total debris-gated, viable, and singlet cells quantified per sample in the brainstem. **(e)** Representative gating strategy for cells acquired in day 5 HSV-infected brainstems, following a similar strategy as described in C. **(f)** Total singlet, viable and debris-gated cells quantified per sample in the TG. **(g)** Representative gating strategy for cells acquired in day 4 HSV-infected TG.

Supplementary Data File 1. HSV-1-aligned RNA-seq reads.

Sheet 1 (“Viral Raw Counts”) contains raw counts for sequencing read that aligned to the HSV-1 strain 17 reference genome (GenBank: JN555585.1), including 75 viral features/open reading frames (Region), across 19 brainstem samples. Sheet 2 (“Viral CPM”) contains counts per million (CPM) host-aligned reads for these same 75 features and 19 brainstem samples. BS: brainstem, mut: *C307X*, HSV: day 5 post-HSV-1 infection, HI: high-responder, LO: low-responder, NI: non-infected, LAT: latency-associated transcript, IE: immediate-early phase, E: early phase, L: late phase.

Supplementary Data File 2. Differentially expressed gene lists.

Sheet 1 (“45 DEG_C307XNI vs wtNI_Fig2a”) lists 45 DEG by comparing mutant (*C307X* NI) mice (n = 4) vs. wild-type (+/+ NI) mice (n = 4) at steady-state, corresponding to data presented in Fig. 2a and Supplementary Fig. 3a. Sheet 2 (“163 DEG_wtHI vs wtNI_Fig3a”) lists 163 DEG by comparing wild-type (+/+ HI) mice (n = 3) at day 5 post-HSV-1 infection vs. wild-type (+/+) NI mice (n = 4) at steady-state, corresponding to data presented in Fig. 3a. Sheet 3 (“256 DEG_C307XHI vs wtNI_Fig3b”) lists 256 DEG by comparing mutant (*C307X* HI) mice (n = 3) at day 5 post-HSV-1 infection vs. wild-type (+/+) NI mice (n = 4) at steady-state, corresponding to data presented in Fig. 3b. Sheet 4 (“51 DEG_C307XHI vs wtHI_Fig4a”) lists 51 DEG by comparing mutant (*C307X* HI) mice (n = 3) vs. wild-type (+/+ HI) mice (n = 3) at day 5 post HSV-1-infection, corresponding to data presented in Fig. 4a. Sheet 5 (“331 DEG_HSV HI vs NI_SFig3b”) lists 331 DEG by comparing all high-responding mice (HSV HI) mice (n = 6) at day 5 post-HSV-1 infection vs. all non-infected mice (NI) mice (n = 8), irrespective of genotype group; these data correspond with Supplementary Fig. 3b. All DEG listed above met a fold-change cut-off of +/- 1.5 and a q-value cut-off of < 0.05 (Benjamini-Hochberg adjusted).

Supplementary Data File 3. Enriched gene ontology (GO) terms.

Sheet 1 (“GO terms_wt_Fig3f”) lists all enriched GO terms in wild-type (+/+ HI) mice (n = 3) vs. +/+ LO mice (n = 2) at day 5 post-HSV-1 infection; select GO terms are summarized in Fig. 3f. Sheet 2 (“GO terms_C307X_Fig3f”) lists all enriched GO terms in mutant (*C307X* HI) mice (n =

3) vs. *C307X* LO mice (n = 3) at day 5 post-HSV-1 infection; select GO terms are summarized in Fig. 3f. Sheet 3 ("GO terms_C307XHI vs wtHI_Fig4b") lists all enriched GO terms in mutant (*C307X* HI) mice (n = 3) vs. wild-type (+/+ HI) mice (n = 3) at day 5 post-HSV-1 infection; select GO terms are summarized in Fig. 4b. All enriched GO terms met a nominal p-value cut-off of < 0.05.

Supplementary Data File 4. Lists of enriched gene sets by gene set enrichment analysis (GSEA).

Sheet 1 ("GSEA_Enriched in C307X NI_Fig2d") lists 66 gene sets (from the MSigDB v6.2 Curated Gene Sets (C2) collection) that were enriched in mutant (*C307X* NI) mice (n = 4) compared to wild-type (+/+ NI) mice (n = 4) at steady-state, as shown in Fig. 2d. Sheet 2 ("GSEA_Enriched in wtNI_Fig2d") lists 80 gene sets (from the MSigDB v6.2 Curated Gene Sets (C2) collection) that were enriched in wild-type (+/+ NI) mice (n = 4) compared to mutant (*C307X* NI) mice (n = 4) at steady-state, as shown in Fig. 2d. Sheet 3 ("GSEA_Enriched in C307X HI_Fig4c") lists the top 200 enriched gene sets in mutant (*C307X* HI) mice (n = 3) compared to wild-type (+/+ HI) mice (n = 3) at day 5 post-HSV-1 infection, as shown in Fig. 4c; these gene sets were taken from the MSigDB v6.2 Immunological Signatures (C7) collection. For all data sets, enriched gene sets met a Bonferroni-Hochberg-adjusted q-value cut-off < 0.05, and were clustered hierarchically according to the expression of leading genes in each gene set. Enriched gene sets are listed in the same order in which they appear in the heatmaps in Fig. 2d and Fig. 4c.
